# Conventional laparoscopy versus Hugo RAS in general surgery: A systematic review and meta-analysis of perioperative outcomes

**DOI:** 10.1016/j.sipas.2026.100371

**Published:** 2026-11-06

**Authors:** Sascha Vaghiri, Alireza Pandkhahi, Dimitrios Prassas, Emmanuella Habib, Norma Schmitz-Conti, Wolfram Trudo Knoefel, Andrea Alexander

**Affiliations:** a Department of Surgery (A), Heinrich-Heine-University, Medical Faculty and University Hospital Duesseldorf, Moorenstr. 5, Bldg. 12.46, 40225, Duesseldorf, Germany; b Medical Research School, Medical Faculty, Heinrich-Heine-University Duesseldorf, Germany

**Keywords:** Robotic surgery, Hugo RAS, Laparoscopy, General surgery, Postoperative complications

## Abstract

**Introduction:**

The Hugo Robotic-Assisted Surgery (RAS) system is a relatively new robotic platform increasingly used in minimally invasive surgery. However, comparative data on its clinical outcomes versus those of conventional laparoscopy in general surgery remain limited. We performed a systematic review and meta-analysis to compare perioperative and postoperative outcomes between Hugo RAS and conventional laparoscopy.

**Methods:**

PubMed, Cochrane CENTRAL, Scopus, and Google Scholar were searched from inception to 16 July 2026. Comparative studies of adult patients undergoing minimally invasive general surgery using Hugo RAS or conventional laparoscopy were included. The primary outcomes were overall postoperative complications and major complications (Clavien–Dindo grade ≥ III). Secondary outcomes included operative time, length of hospital stay, conversion to open surgery, postoperative ileus, anastomotic leakage, reoperation, and readmission rates. Pooled odds ratios (ORs) and standardized mean differences (SMDs), with 95% confidence intervals (CIs), were calculated. Risk of bias was assessed using ROBINS-I, and certainty of evidence was evaluated using GRADE.

**Results:**

The search identified 4158 records, of which three retrospective and one prospective comparative studies, involving a total of 898 patients, met the inclusion criteria. Overall, 161 patients underwent Hugo RAS procedures and 737 underwent conventional laparoscopic surgery. The rate of overall postoperative complications was similar between the two groups (OR = 0.80; 95% CI, 0.49–1.31; *p* = 0.38; I² = 12%). However, major postoperative complications (Clavien–Dindo grade ≥ III) were significantly less frequent in the Hugo RAS group (OR 0.31, 95% CI 0.10–0.92; *p* = 0.04; I²=0%), although the certainty of evidence was low. No significant differences were found for other outcomes of interest.

**Conclusion:**

Hugo RAS appears to be a safe and technically feasible alternative to conventional laparoscopy in minimally invasive general surgery, with similar overall postoperative outcomes. The lower rate of major postoperative complications observed with Hugo RAS is encouraging, but should be interpreted cautiously because the available evidence is based on only four non-randomized studies and is subject to methodological limitations and clinical heterogeneity. Further prospective multicenter studies are needed to determine whether these findings are consistent across different surgical procedures.

## Introduction

Minimally invasive surgery has been revolutionized by the introduction of robotic systems. Compared with conventional laparoscopy, robotic surgery provides enhanced dexterity, high-definition visualization, and improved ergonomics, facilitating precise dissection and resection in confined anatomical regions and in fragile or inflamed tissues [[1], [2], [3]]. These advantages have led to its widespread adoption across multiple surgical specialties. Several high-quality randomized controlled trials (RCTs) have demonstrated that robotic surgery is technically safe and feasible and is associated with lower surgical morbidity, faster recovery, and better oncological outcomes compared with laparoscopy [[4], [5], [6]]. The expanding range of robotic surgical platforms, together with the dominance of the well-established da Vinci surgical system, economic considerations, and substantial acquisition and maintenance costs, may complicate both the adoption of robotic surgery and the transition to alternative platforms by making selection of the most appropriate system more complex [7,8]. The Hugo™ Robotic-Assisted Surgery (RAS) system (Medtronic, Minneapolis, MN, USA) became commercially available in 2021 as a cost-effective alternative to the da Vinci robot and is characterized by innovative technical features such as a flexible modular cart system, an open console configuration, and high-definition three-dimensional visualization [[9], [10], [11]]. The Hugo RAS system is now being increasingly implemented in a wide range of surgical specialties, including colorectal, general, and urologic surgery [8,[12], [13], [14]].

However, there is a significant paucity of studies comparing outcomes of conventional laparoscopy and the Hugo RAS platform in general surgery. To address this concern, we performed a systematic review and meta-analysis comparing pooled perioperative outcomes of general surgery procedures performed using conventional laparoscopy and the Hugo RAS system.

## Materials and methods

This systematic review and meta-analysis was structured according to the PRISMA (Preferred Reporting Items for Systematic Reviews and Meta-analyzes) guidelines and followed the methodology outlined in the Cochrane Handbook for Systematic Reviews of Interventions [15,16]. The study was registered in PROSPERO (CRD420261528471).

### Search strategy

The primary search focused on studies comparing perioperative outcomes between the Hugo RAS platform and conventional laparoscopy in general surgery, including colorectal, hernia surgery, and cholecystectomy). Two authors (S.V. and A.P.) independently performed the systematic literature search by manually screening titles and abstracts in the PubMed (MEDLINE), Cochrane Central Register of Controlled Trials (CENTRAL), Scopus, and Google Scholar databases. No automated screening tools were used for eligibility decisions. The systematic literature search was performed across relevant electronic databases from inception to 16 July 2026 and primarily included articles published in English.

The search strategy combined the following keywords: "Hugo", "Hugo RAS", "robotic surgery", "robotic-assisted surgery", "RAS", "laparoscopy", "laparoscopic surgery", "colorectal surgery", "colorectal cancer", "hernia", "inguinal hernia", and "general surgery". Suitable articles were then evaluated by full-text review. Additionally, the reference lists of all retrieved articles were screened for additional relevant studies.

### Study selection

We used the PICO framework to define the eligibility criteria and guide the identification and selection of eligible studies as follows:

P (Population): Patients undergoing minimally invasive general surgery (e.g. colorectal, hernia, cholecystectomy, etc.).
I (Intervention): Surgery performed using conventional laparoscopy.
C (Comparison): Surgery performed using the Hugo RAS platform.
O (Outcomes): Postoperative complications and major morbidity (according to the Clavien-Dindo classification [17]) as the primary outcomes, reoperation and readmission rates, length of hospital stay (LOS), duration of surgery (min), conversion to open surgery, and pathological and functional outcomes (defined as the secondary outcomes).

No restrictions regarding study design or sample size were imposed. The following exclusion criteria were applied: 1) inapplicable sources such as non-comparative studies, case reports, technical reports, editorials, narrative reviews, gray literature, and unpublished literature; 2) duplicate reports or reports from potentially overlapping patient cohorts when outcomes for distinct patients could not be extracted separately; 3) non-adult patients (aged < 18 years); 4) emergency surgical procedures; 5) inability to calculate odds ratios (ORs) or standardized mean differences (SMDs) with 95% confidence intervals (CIs) for the reported outcomes of interest; and 6) studies comparing robotic platforms other than Hugo RAS with laparoscopy.

### Data extraction

Two authors (S.V. and E.H.) independently collected all relevant available data from studies meeting the inclusion criteria. In addition to the predefined outcomes of interest, data regarding study characteristics (authors, country of origin, study design, population, and study period and follow-up duration), patient demographics (age, sex, body mass index [BMI], American Society of Anesthesiologists [ASA] score, and comorbidities), disease characteristics, surgical indications, types of procedures, and operative characteristics were extracted.

### Risk of bias and certainty assessment

The ROBINS-I tool [18] was used to assess the risk of bias in all non-randomized studies, as endorsed by the Cochrane Handbook for Systematic Reviews of Interventions [16]. Two authors (S.V. and N.S.C.) independently evaluated each study across seven specific domains to classify the risk of bias. The GRADE (Grading of Recommendations, Assessment, Development, and Evaluation) criteria [19] were applied to assess the certainty of evidence for the primary and secondary outcomes. Any disagreements during study selection, data extraction, or risk-of-bias assessment stages were resolved through discussion and consensus between the two reviewers or, if necessary, through consultation with a third senior author (A.A.).

### Statistical analysis

Statistical analysis was performed using RevMan software (version 5.3; Copenhagen: The Nordic Cochrane center, The Cochrane Collaboration, 2014). Summary treatment-effect estimates with 95% confidence intervals (CIs) were calculated with odds ratios (ORs) for dichotomous outcomes and standardized mean differences (SMDs) for continuous outcomes. Pooled effect estimates were calculated using fixed- or random-effects models, as appropriate, according to current methodological guidance. Dichotomous outcomes were analyzed using the Cochran–Mantel–Haenszel method, and continuous outcomes were analyzed using the inverse-variance method. Where necessary, medians and (interquartile) ranges were converted into means and standard deviations using the methods proposed by Hozo et al. and Luo et al. [20,21]. Statistical heterogeneity was assessed using Cochran's Q test (chi-squared test; χ²) and the I² statistic. Accordingly, I² values of 0–40% might not be important (low heterogeneity), 30–60% may represent moderate heterogeneity, 50–90% may represent substantial heterogeneity, and >75% may represent considerable heterogeneity [16]. If substantial heterogeneity (I^2^ > 50%) was identified, potential sources of heterogeneity were explored through leave-one-out analysis. A narrative review was provided for outcomes for which quantitative synthesis was not possible. Publication bias was not assessed because of the limited number of included studies, as recommended. Statistical significance was defined as *p* < 0.05.

## Results

### Study and patient summary

The literature search across the designated databases yielded 4158 records. After screening according to the eligibility criteria, 14 full-text articles underwent critical evaluation. Of these, four studies [[22], [23], [24], [25]] met the inclusion criteria and were included in the final analysis (Fig. 1). At full-text assessment, Rottoli et al. [26] was excluded from quantitative synthesis because it originated from the same center as Violante et al. [24], with potentially overlapping patients with ileocecal Crohn’s disease. Although Rottoli et al. also reported other inflammatory bowel disease operations, comparative outcomes for these distinct procedures could not be extracted separately for the pooled analyzes. Of note, the study by Violante et al. [24] was the most recent publication from this group and included a substantially larger patient cohort.

**Fig. 1:**
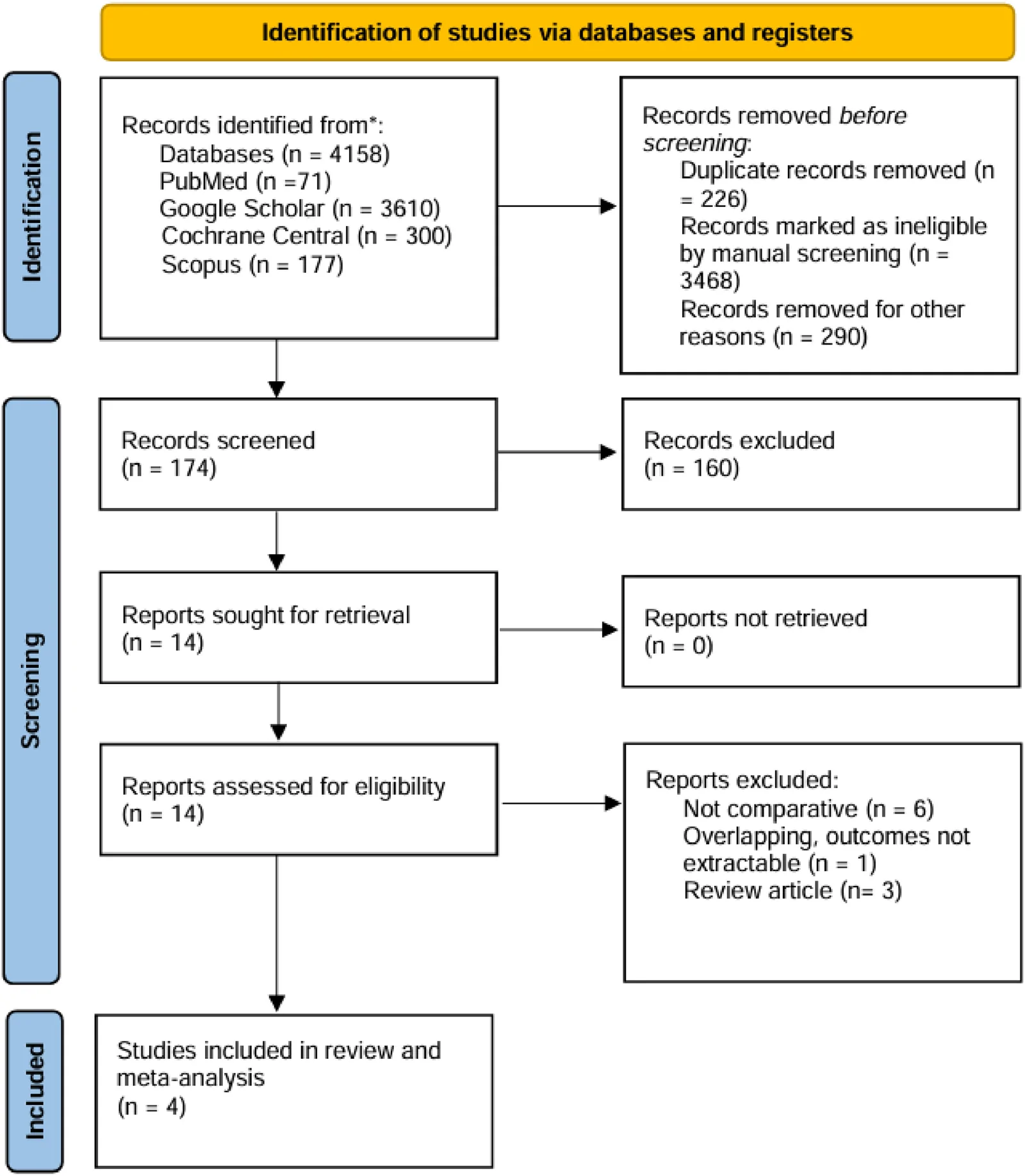
PRISMA flowchart of study search and selection.

Two studies originated from Spain [23,25], and two were conducted in Italy [22,24]. In the studies by Arroyo et al. [23] and Calini et al. [22] patients underwent oncological colorectal surgery. Violante et al. [24] included 685 patients with ileocecal resection for Crohn’s disease and Caruso et al. [25] compared outcomes between Hugo RAS and laparoscopic bilateral transabdominal preperitoneal inguinal hernia repair (TAPP). The postoperative follow-up period ranged from one month in Violante et al. [24] to 24 months in the study by Caruso et al. [25]. In the pooled analysis, 161 patients were included in the Hugo RAS group, and 737 underwent laparoscopic surgery. Study and patient characteristics are summarized in Table 1, Table 2. All non-randomized studies were retrospective in design, with the exception of one prospective study [25]. The risk of bias ranged from moderate to serious (Table 1). Propensity score matching (PSM) was performed in Violante et al. [24]. The detailed ROBINS-I assessment for each domain is provided in Fig. supp. 1.

**Table 1:** Study characteristics and protocols.

Author	Year	Origin	Study period	Study design	Total sample size	Exclusion criteria	Operative Indication	Follow-up period	Endpoints	Risk of bias (ROBIS-I)
Arroyo et al. [23]	2026	Spain	January 2023- February 2024	Retrospective, single center	40	Emergency surgery, recurrent rectal cancer, multivisceral resection, and previous pelvic surgery	Rectal cancer	6 months	Postoperative complications, hospital stay, LARS, and residue of the disease	Moderate
Caruso et al. [25]	2026	Spain	February 2023- November 2024	Prospective, single-center	64	Patients <18 years, open repair, and treated for unilateral hernias	Bilateral inguinal hernia repair	16–36 months	Total operation cost, postoperative complications, pain score, hospital stays, and operation times	Serious
Violante et al. [24]	2026	Italy	January 2003- June 2025	Retrospective, single-center, PSM	685	Emergency surgery, missing perioperative data	Ileocecal Crohn’s Disease	30-days	30-day postoperative complications, operative time, conversion rate, intraoperative complications, length of hospital stay, and readmission rate	Moderate
Calini et al. [22]	2025	Italy	April 2023-October 2024	Retrospective, single-center	109	Emergency surgery, patients <18 years, with any histologic diagnosis other than colorectal adenocarcinoma	Colorectal Cancer	30-days	30-day overall postoperative complication, and length of hospital stay	Moderate

**Table 2:** Demographic data and characteristics of the included patients.

Author	Groups	Number of included patients	Age	Gender (M/F)	Body Mass Index (kg/m2)	ASA score	Type of surgery	Previous Abdominal Surgery	Tumor stage
Arroyo et al. 2026 [23]	Hugo RAS	15	65+−21	M:12, F:3	NA	I-II: 8 III:6 IV:1	Rectal resection:15	NA	I:4 II:5 III:6
	Laparoscopy	25	65.3+−11.7	M:18, F:7	NA	I-II:15 III:10	Rectal resection:5	NA	I:14 II:3 III:8
Caruso et al. 2026 [25]	Hugo RAS	32	70	M:29, F:3	25.8	1.9	Bilateral TAPP*:32	NA	NA
	Laparoscopy	32	64,7	M: 31, F: 1	24.9	2.1	Bilateral TAPP:32	NA	NA
Violante et al. 2026 [24]	Hugo RAS	62	41.83+- 20.74	M:31, F: 31	21.93+- 3.78	I-II: 51 III:10	Ileocecal resection:62	19	NA
	Laparoscopy	623	36.67+- 17.05	M:323, F:300	21.33+- 3.7	I-II: 536 III:76 IV:1	Ileocecal resection:623	165	NA
Calini et al. 2025 [22]	Hugo RAS	52	67+−4.21	M:28, F: 24	26+−1.33	I-II: 29 III:20 IV:3	Colorectal cancer resections:52	23	0:3 I:22 II:15 III:8 IV:4
	Laparoscopy	57	68+−3.49	M:31, F: 26	25+−1.74	I-II: 29 III:28	Colorectal cancer resections:57	26	0:4 I:14 II:20 III:12 IV:7

NA: Not available, RAS: Robotic Assisted Surgery, TAPP: Transabdominal Preperitoneal.

#### Meta-analyzed outcomes

The overall rate of postoperative complications was reported in three studies [[23], [24], [25]] comprising 109 patients in the Hugo RAS group and 680 patients in the laparoscopy group. Meta-analysis demonstrated no significant difference in overall complication rates between Hugo RAS and laparoscopy (OR 0.80, 95% CI: 0.49–1.31; *p* = 0.38; I² = 12%) (Fig. 2a). However, in terms of major morbidity, a significantly lower rate of Clavien–Dindo grade ≥ III complications was observed in four studies [[22], [23], [24], [25]] in the Hugo RAS group compared with the laparoscopic group (OR 0.31, 95% CI: 0.10–0.92; *p* = 0.04; I² = 0%) (Fig. 2b). In the pooled analysis, no significant differences were observed between the two study cohorts in other outcomes, including postoperative ileus, anastomotic leak, ostomy formation, reoperation, and the total number of harvested lymph nodes (Table 3). Postoperative readmissions rates were reported in only two studies [22,24]. Although the readmission rates were not significantly different between Hugo RAS and laparoscopy, a trend toward fewer postoperative readmissions was observed favoring the Hugo RAS platform. The certainty of evidence, assessed using GRADE, was rated as low to very low (Table supp. 1).

**Fig. 2:**
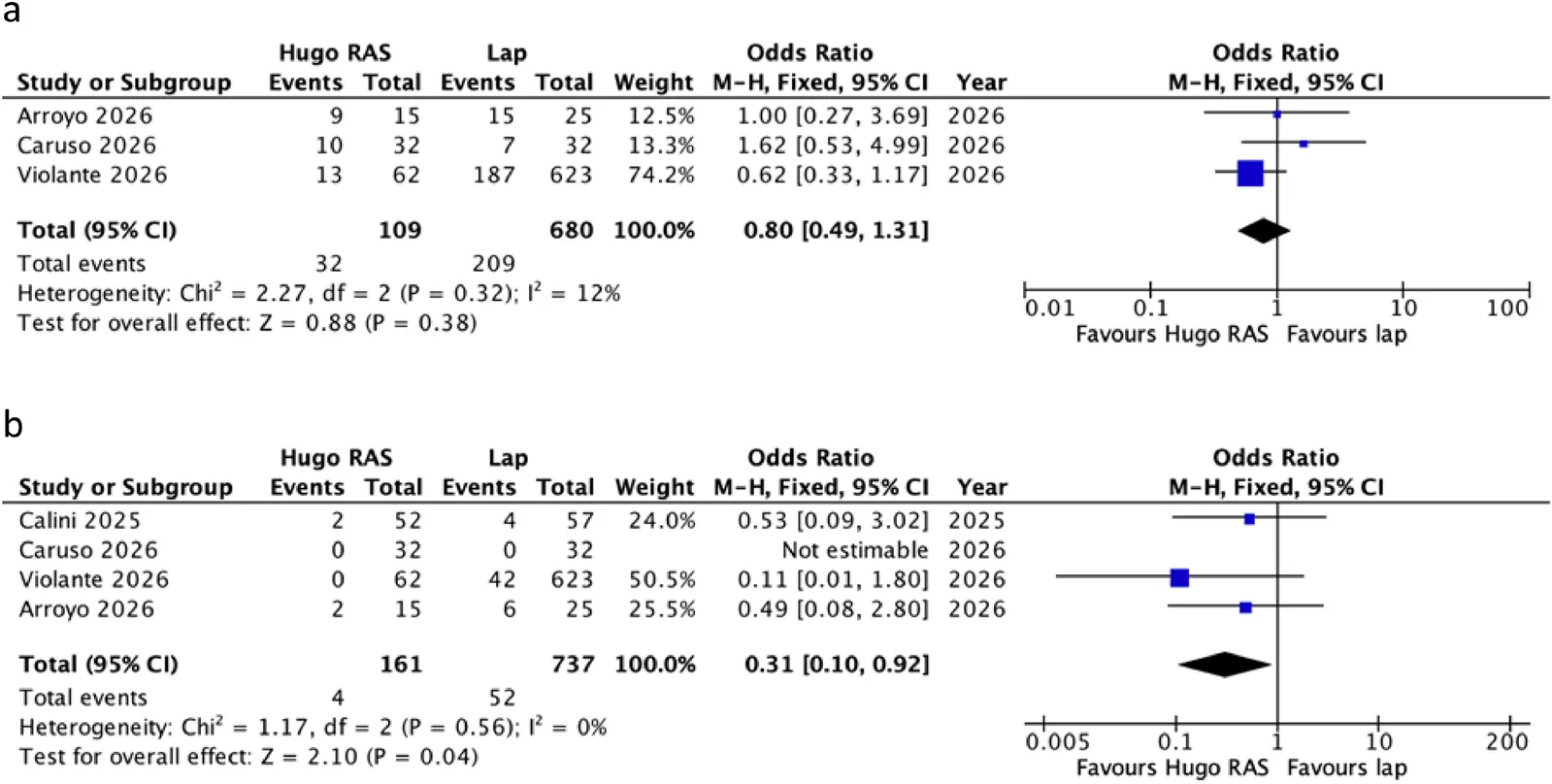
Forest plots for the main outcomes, Hugo RAS versus laparoscopy: a) overall complications, b) major morbidity.

**Table 3:** Pooled analysis of secondary outcomes.

		No. of included patients				Heterogeneity Level	
Outcomes	No. of included studies	Hugo RAS	Laparoscopy	SMD/OR [95% CI]	P-value	I² (%)	P-value
Ileus	3 [22, 23, 25]	99	114	0.37 [0.06–2.39]	0.30	0	0.98
Anastomotic leak	2 [22, 23]	67	82	0.35 [0.05–2.22]	0.26	0	0.63
Ostomy formation	2 [22, 24]	114	680	0.45 [0.18–1.16]	0.10	0	0.32
Readmission	2 [22, 24]	114	680	0.55 [0.29–1.07]	0.08	0	0.36
Reoperation	3 [23, 24, 25]	109	680	0.35 [0.06–2.10]	0.25	31	0.23
Total number of harvested lymph nodes	2 [22, 23]	67	79	−0.08 [−0.41–0.24]	0.61	0	0.37

#### Descriptive outcomes and non-meta-analyzed parameters

The rate of conversion to open surgery was reported in 898 patients across both cohorts (Hugo RAS: n = 161; laparoscopy: n = 737). No conversions were documented in the studies by Arroyo et al. [23] and Caruso et al. [25]. The conversion rate in Calini et al. [22] was 6% in the Hugo RAS cohort and 2% in the laparoscopic group, respectively (*p* = 0.34). In contrast, Violante et al. [24] reported a significantly higher conversion rate (14.8%) associated with laparoscopy compared with 1.7% for Hugo RAS procedures (*p* < 0.001). Duration of surgery was reported in three studies, with Caruso et al. [25] demonstrating significantly longer procedural duration in the Hugo RAS group whereas the studies by Violante et al. [24] and Calini et al. [22] found no significant differences in operative time between the two cohorts . The pooled results showed substantial heterogeneity, precluding meaningful subgroup analysis. Length of hospital stay was reported in all four studies, with conflicting results. In the study by Arroyo et al. [23], the mean hospital stay was significantly longer in the laparoscopic group (7.6 vs. 4.1 days, *p* = 0.038). This was confirmed in Violante et al. [24] with a hospital stay of 13.7 days for laparoscopic surgery and 9.7 days after Hugo RAS resections (*p* = 0.003). Calini et al. [22] and Caruso et al. [25] found no statistically significant difference in the length of hospital stay between the two approaches. Functional outcomes at six-month follow-up were described in detail in the study by Arroyo et al. [23] after rectal cancer surgery. Hugo RAS interventions were significantly associated with a lower mean LARS score (9.08 vs. 19.2, *p* = 0.008), lower pain levels (71%vs. 93%, *p* = 0.0019), and better sexual function in male patients (*p* = 0.045). In Caruso et al. [25] chronic postoperative pain was documented in 6.3% of patients after laparoscopic TAPP versus 0% after Hugo RAS TAPP (*p* = 0.076). The mean total cost of bilateral robotic hernia surgery was €4365.80 in comparison to €1690.54 in the laparoscopic group mainly attributable to a mean robotic usage fee of €2500.00 [25].

## Discussion

This systematic review and meta-analysis is, to the best of our knowledge, the first quantitative synthesis comparing the Hugo RAS platform with conventional laparoscopy in general surgical procedures. Pooled data from four retrospective comparative studies involving 898 patients indicate that the Hugo RAS system appears technically feasible and clinically safe for minimally invasive general surgery. Although overall postoperative morbidity, reoperation, readmission, postoperative ileus, anastomotic leakage, and oncological outcomes were similar between the two approaches, the Hugo RAS platform was associated with a significantly lower incidence of major postoperative complications (CD ≥ III).

The observed reduction in major postoperative complications is clinically relevant, as these events substantially influence postoperative recovery and patient prognosis [17]. Interestingly, while the overall postoperative complication rate remained comparable between the two groups, the incidence of major complications was consistently lower in the Hugo RAS groups across the included studies. This observation suggests that robotic assistance may influence the severity, rather than the overall frequency, of postoperative adverse events. Arroyo et al. [23] reported lower rates of major complications and anastomotic leakage, together with improved postoperative functional outcomes following Hugo RAS-assisted rectal resection, despite including a higher proportion of patients with advanced tumors. Likewise, Violante et al. [24] demonstrated lower conversion and 30-day readmission rates after Hugo RAS-assisted ileocecal resection for Crohn's disease, while Calini et al. [22] showed that experienced laparoscopic surgeons were able to transition safely to the Hugo platform without compromising perioperative outcomes.

These observations should not be considered evidence that the Hugo RAS platform is inherently superior to conventional laparoscopy. Nevertheless, they are biologically plausible. The Hugo RAS system contains several technical features, including an open surgical console and four independently mobile robotic-arm carts that enable flexible docking and facilitate communication between the console surgeon and bedside assistant [8,9,12,[27], [28], [29]]. Together with the established advantages of robotic-assisted surgery, these characteristics may facilitate more precise dissection during technically demanding procedures and could partly account for the lower incidence of major postoperative complications observed in the present analysis [3,9,12]. Given the retrospective nature of the available evidence, this interpretation should be regarded as hypothesis-generating and requires confirmation in adequately powered prospective studies.

In contrast to the reduction in major postoperative complications, operative time was comparable between Hugo RAS and conventional laparoscopy. This is not unexpected, as operative duration during the early adoption of a new robotic platform is influenced by several factors, including procedure complexity, docking strategy, institutional experience, and the surgical learning curve. Similar patterns have been reported in both in the comparative studies included in the present analysis and in subsequent Hugo RAS-specific series, where operative efficiency improved with increasing experience while perioperative outcomes remained unchanged [12,[29], [30], [31], [32]]. Of note, in Arroyo et al. [23] and Calini et al. [22] the operating surgeons were inexperienced in robotic surgery before implementation of the Hugo RAS system. Beyond conventional perioperative outcomes, Hugo RAS may also have a positive impact on postoperative functional recovery. Arroyo et al. [23] reported improved bowel and sexual function following Hugo-assisted rectal resection, together with lower rates of major complications and anastomotic leakage, despite treating a higher proportion of patients with locally advanced rectal cancer. However, these findings are based on a single comparative study and should be confirmed in larger prospective investigations. Beyond its clinical outcomes, the Hugo RAS platform may also contribute to the broader adoption of robotic surgery by increasing competition and improving accessibility. Nevertheless, evidence regarding its economic value remains limited, and further cost-effectiveness studies are necessary to define its role in routine general surgical practice [8,12,27,28].

To the best of our knowledge, this study represents the first quantitative synthesis comparing Hugo RAS with conventional laparoscopy in general surgery. By including only comparative studies and systematically assessing methodological quality, this review provides a comprehensive evaluation of the currently available clinical evidence. The results should be regarded as exploratory and hypothesis-generating rather than definitive estimates applicable across surgical indications. In particular, we emphasize that the pooled results primarily provide an overall description of the currently available evidence regarding the Hugo RAS system and should not be interpreted as evidence of equivalent outcomes across different procedures or patient populations. This study has several limitations. All included studies were non-randomized, had small sample sizes, and were subject to selection bias. Moreover, only four comparative studies were available, and heterogeneity in surgical procedures and surgeon experience may have influenced the pooled estimates. In addition, long-term functional, oncological, and economic outcomes remain insufficiently investigated. The presented results could be valuable, taken into consideration the worldwide adoption of robotic surgery and the implementation of a new robotic platform in institutions, where still minimally invasive surgery is performed by conventional laparoscopy.

## Conclusion

In conclusion, the available evidence suggests that the Hugo RAS platform is a safe and feasible option for general surgery when implemented in experienced minimally invasive surgical centers, without compromising perioperative or short-term oncological outcomes. Although Hugo RAS was associated with a lower incidence of major postoperative complications in our meta-analysis, these findings should be interpreted with caution in light of the limited number of non-randomized comparative studies, the heterogeneity of the included procedures, and the consequent lack of statistical power. Larger randomized multicenter studies are required to determine whether the observed clinical advantages can be consistently reproduced across different surgical procedures and healthcare settings.

## Informed consent

For this type of study, informed consent was not required.

## CRediT authorship contribution statement

**Sascha Vaghiri:** Writing – original draft, Formal analysis, Data curation, Conceptualization. **Alireza Pandkhahi:** Writing – original draft, Formal analysis. **Dimitrios Prassas:** Formal analysis, Data curation, Conceptualization. **Emmanuella Habib:** Methodology, Data curation. **Norma Schmitz-Conti:** Writing – review & editing, Methodology. **Wolfram Trudo Knoefel:** Writing – review & editing, Supervision. **Andrea Alexander:** Writing – original draft, Conceptualization.

## Declaration of competing interest

The authors declare that they have no known competing financial interests or personal relationships that could have appeared to influence the work reported in this paper.

## Data Availability

The datasets used and/or analyzed during the current study are available from the corresponding author upon reasonable request.

## Appendix Supplementary materials

**Supplementary Table 1.** GRADE certainty assessment for significant outcomes.
