# Supplementary material for: Conventional laparoscopy versus Hugo RAS in general surgery: A systematic review and meta-analysis of perioperative outcomes
Source: Surg Pract Sci. 2026 Nov 6;27:100371. doi: 10.1016/j.sipas.2026.100371 (PMC13770604; doi:10.1016/j.sipas.2026.100371)
Supplement: Supplementary file 1 [file mmc1.docx]

**Fig. suppl. 1:** Risk of bias evaluation according to ROBINS-I

| Table Suppl. 1 GRADE certainty assessment for significant outcomes | | | | | | | | | | |
| --- | --- | --- | --- | --- | --- | --- | --- | --- | --- | --- |
| Outcomes | No. of studies | **No. of included patients** | | SMD/OR [95 % CI] | **Quality assessment** | | | | | Quality |
|  |  | Hugo RAS | Laparoscopy |  | Risk of bias^a^ | Inconsistency | Indirectness | Imprecision | Publication bias |  |
| Major morbidity | 4 [22-25] | 161 | 737 | 0.31 [0.10-0.92] | Serious | Not serious | No indirectness | No imprecision | NA | Low |
| Overall morbidity | 3 [23,24,25] | 109 | 680 | 0.80 [0.49–1.31] | Serious | Not serious | No indirectness | Serious | NA | Very low |
| Ileus | 3 [22, 23, 25] | 99 | 114 | 0.37 [0.06-2.39] | Serious | Not serious | Serious | Serious | NA | Very low |
| Anastomotic leak | 2 [22, 23] | 67 | 82 | 0.35 [0.05-2.22] | Serious | Not serious | No indirectness | Serious | NA | Very low |
| Ostomy formation | 2 [22, 24] | 114 | 680 | 0.45 [0.18-1.16] | Serious | Not serious | No indirectness | Serious | NA | Very low |
| Readmission | 2 [22, 24] | 114 | 680 | 0.55 [0.29-1.07] | Serious | Not serious | No indirectness | Serious | NA | Very low |
| Reoperation | 3 [23, 24, 25] | 109 | 680 | 0.35 [0.06-2.10] | Serious | Not serious | No indirectness | Serious | NA | Very low |
| Total number of harvested lymph nodes | 2 [22, 23] | 67 | 79 | -0.08 [-0.41-0.24] | Serious | Not serious | No indirectness | Serious | NA | Very low |
| CI: Confidence interval, NA: Not applicable, OR: Odds ratio, SMD: Standardized mean deviation  ^a^ Risk of bias assessed using the ROBINS-I tool | | | | | | | | | | |
| Table Suppl. 1 GRADE certainty assessment for significant outcomes | | | | | | | | | | |
| Outcomes | No. of studies | **No. of included patients** | | SMD/OR [95 % CI] | **Quality assessment** | | | | | Quality |
|  |  | Hugo RAS | Laparoscopy |  | Risk of bias^a^ | Inconsistency | Indirectness | Imprecision | Publication bias |  |
| Major morbidity | 4 [22-25] | 161 | 737 | 0.31 [0.10-0.92] | Serious | Not serious | No indirectness | No imprecision | NA | Low |
| Overall morbidity | 3 [23,24,25] | 109 | 680 | 0.80 [0.49–1.31] | Serious | Not serious | No indirectness | Serious | NA | Very low |
| Ileus | 3 [22, 23, 25] | 99 | 114 | 0.37 [0.06-2.39] | Serious | Not serious | Serious | Serious | NA | Very low |
| Anastomotic leak | 2 [22, 23] | 67 | 82 | 0.35 [0.05-2.22] | Serious | Not serious | No indirectness | Serious | NA | Very low |
| Ostomy formation | 2 [22, 24] | 114 | 680 | 0.45 [0.18-1.16] | Serious | Not serious | No indirectness | Serious | NA | Very low |
| Readmission | 2 [22, 24] | 114 | 680 | 0.55 [0.29-1.07] | Serious | Not serious | No indirectness | Serious | NA | Very low |
| Reoperation | 3 [23, 24, 25] | 109 | 680 | 0.35 [0.06-2.10] | Serious | Not serious | No indirectness | Serious | NA | Very low |
| Total number of harvested lymph nodes | 2 [22, 23] | 67 | 79 | -0.08 [-0.41-0.24] | Serious | Not serious | No indirectness | Serious | NA | Very low |
| CI: Confidence interval, NA: Not applicable, OR: Odds ratio, SMD: Standardized mean deviation  ^a^ Risk of bias assessed using the ROBINS-I tool | | | | | | | | | | |
